# Supplementary material for: A wheat cysteine-rich receptor-like kinase confers broad-spectrum resistance against Septoria tritici blotch
Source: Nat Commun. 2021 Jan 19;12:433. doi: 10.1038/s41467-020-20685-0 (PMC7815785; doi:10.1038/s41467-020-20685-0)
Supplement: Supplementary file 1 — Supplementary information [file 41467_2020_20685_MOESM1_ESM.pdf]

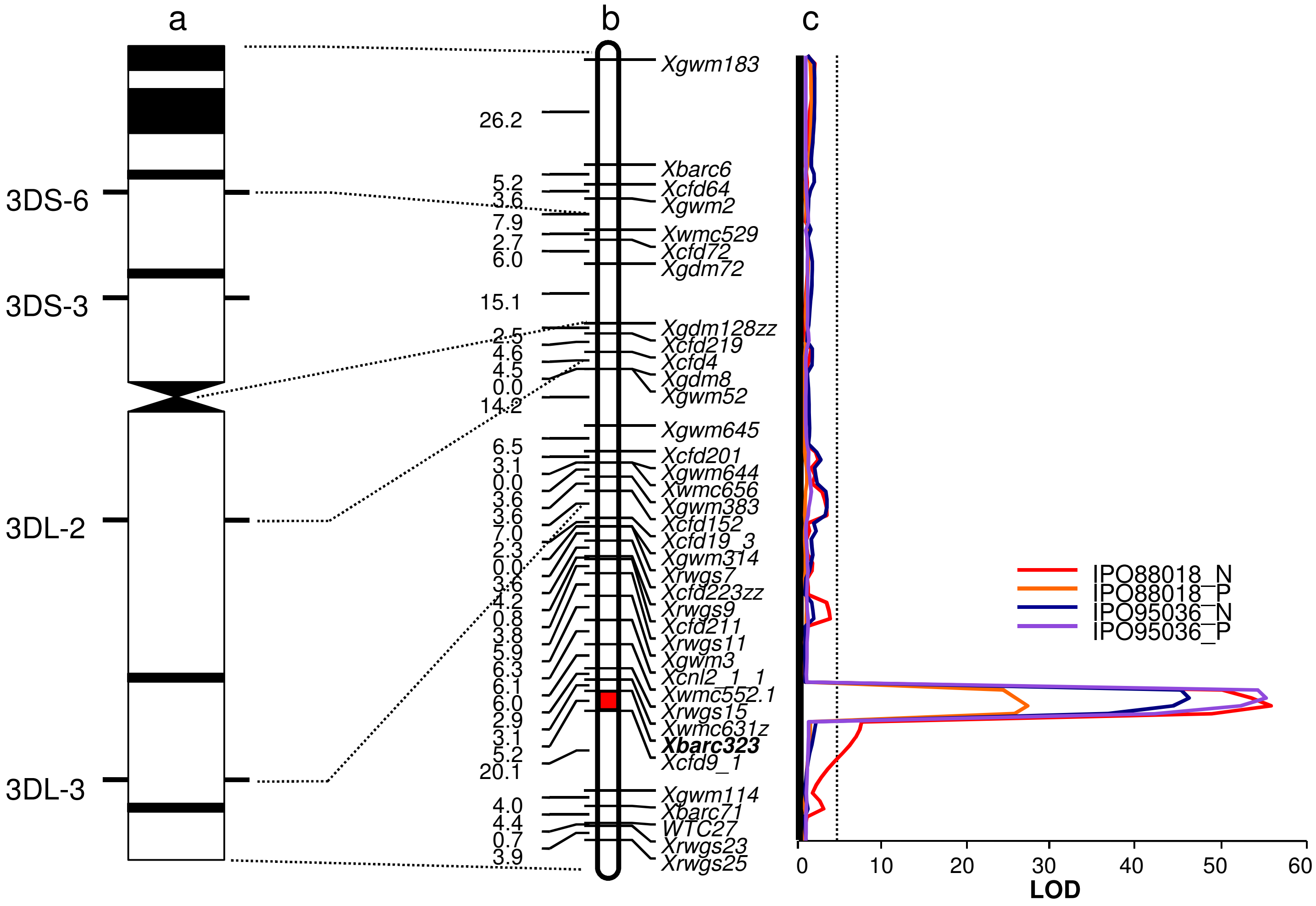

Supplementary Figure 1: Genetic mapping of STB resistance using a bi-parental population derived from the cross between the SHW TA4152-19 and ND495. a – Chinese Spring deletion-based physical map of chromosome 3D. Black boxes indicate chromosome 3D C-banding pattern. Deletion breakpoints are indicated to the left of the map. b – Chromosome 3D linkage map. Distances are indicated on the left in cM and markers are shown along the right. c –LOD values of QTLs identified following phenotypic evaluation of the population with two *Z. tritici* isolates (IPO88018 and IPO95036) and two phenotypic traits, N (necrosis) and P (pycnidia). Comparisons of the markers on the genetic linkage map with their locations on the chromosome 3D physical map indicated that the *Stb16q* locus was located in the most distal deletion bin on chromosome arm 3DL (3DL-3). This first phase of mapping delineated the QTL corresponding to *Stb16q* to a 5.2 cM segment flanked by SSR markers *barc323* and *cfd9* and indicated with a red box.

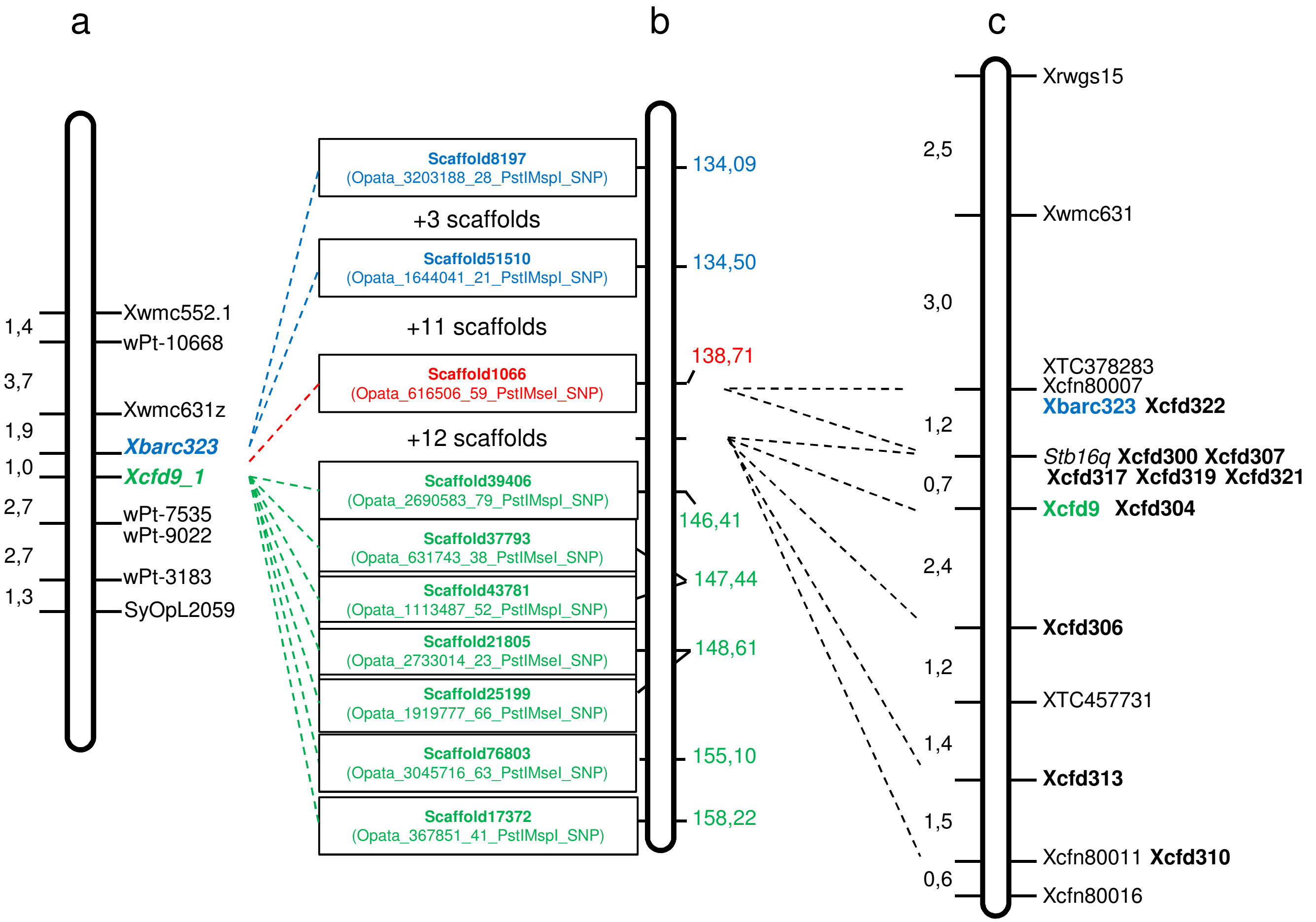

Supplementary Figure 2: Marker enrichment at the *Stb16q* locus. a - Linkage map of chromosome 3D derived from the ITMI population[1] including *Stb16q*-flanking SSR markers *barc323* and *cfd9*. A total of 42 GBS-derived SNP markers were either co-segregating with *barc323* or *cfd9* or present in the interval defined by these two SSR markers (data not shown). b – Physical map of the *Stb16q* locus anchored to the D genome linkage map[2]. Using a BLAST search ten of the 42 SNP markers (indicated on brackets to the left) were anchored to ten scaffold of the D genome. Scaffolds co-segregating with *barc323*, present in the SSRs interval or co-segregating with *cfd9* are indicated in blue, red and green, respectively. Scaffolds carrying no SNP marker but present in the physical interval were not represented. Among these latter, scaffold45305 is present between scaffold1066 and scaffold39406. Distances of the linkage map are indicated on the right in cM. This approach allowed us to delimit *Stb16q* to a physical interval of twenty-six scaffolds (from scaffold51510 to scaffold39406) and 12 cM. SSR markers were designed from all these scaffolds and polymorphic ones between the resistant accession TA4152-19 and the susceptible line ND495 were genetically mapped using the bi-parental DH mapping population derived from the cross between these two accessions. c – Linkage map issued from the DH TA4152-19 × ND495 mapping population. SSR markers derived from the D genome scaffolds are indicated in bold. Markers *cfn* were derived from SNP markers[3]. Markers STS and TC were developed from bin-mapped EST known to map to the most distal bin of chromosome 3D. Distances of linkage maps are indicated in cM.

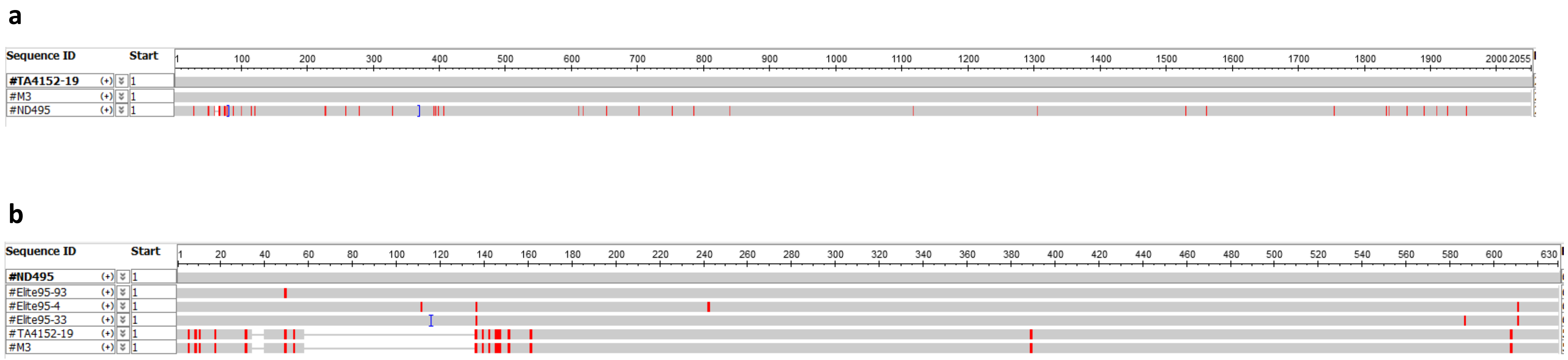

Supplementary Figure 3: Haplotypes of *Crk6* (a) and *Unk1* (b) coding sequences. The different haplotypes are depicted as horizontal grey bars. Vertical red bars and blue bars represent SNP and insertions relative to the master haplotype TA4152-19 (a) and ND495 (b), respectively. Deletions are represented by thin grey horizontal bars. The top scale indicates base pairs. Only haplotypes of SHWs M3 and TA4152-19 and ND495 are represented for *Crk6*. All haplotypes are depicted in Figure 2. The five different haplotypes identified among the 76 SHWs are represented for the *Unk1* gene.

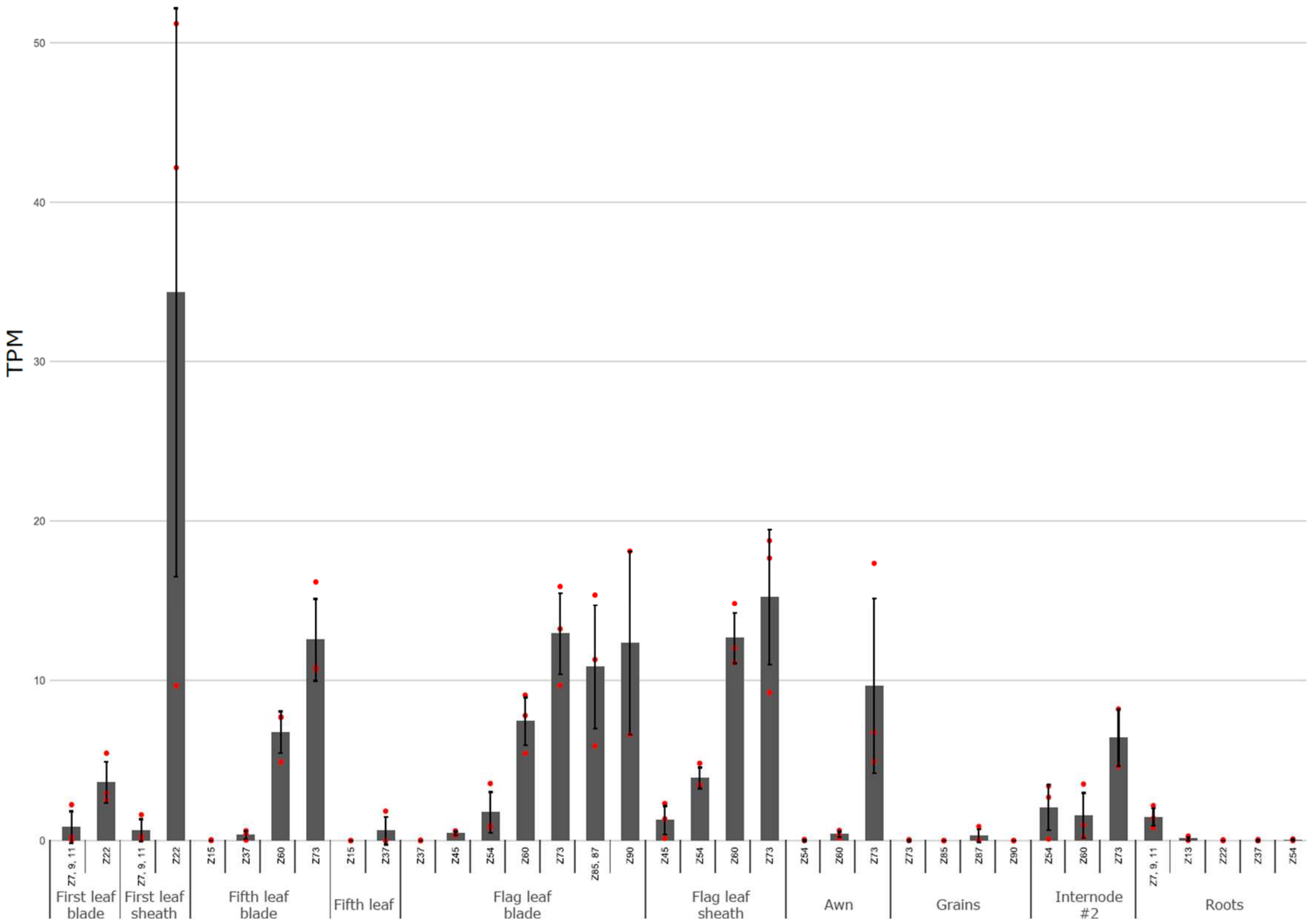

Supplementary Figure 4: *Stb16q* expression in different tissues of spring wheat cultivar Azhurnaya. Transcripts per kilobase millions (TPM) values were obtained from RNA-seq data for *Stb16q* from three biological replicates of five individuals plants each[4]. Error bars represent mean ± s.d. Dots represent individual data points. Growth stages are defined as in Zadocks et al.[5].

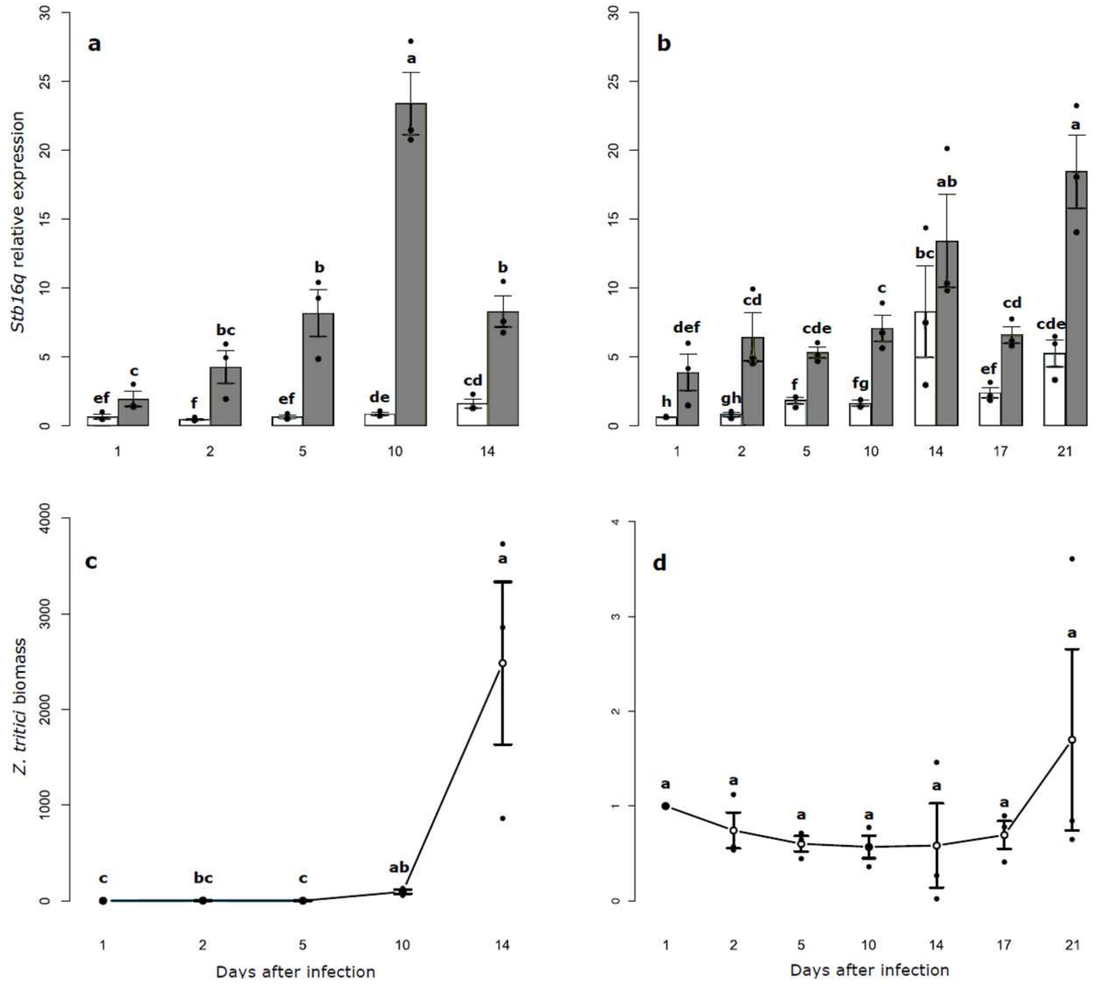

Supplementary Figure 5: *Stb16q* relative expression and *Z. tritici* biomass during a time course infection on wheat. Transcript level of *Stb16q* was evaluated by real-time PCR from the second leaves of the susceptible accession ND495 (a) and of the resistant accession TA4152-19 (b) and after mock inoculation (white bars) or inoculation with *Stb16q* avirulent isolate IPO9415 (grey bars). The Pfaffl method[6] was used to calculate relative expression values compared with time point 0 and normalization was achieved by the wheat phytochelatin gene[7]. *Z. tritici* biomass was measured by real-time PCR on the *Z. tritici* β-tubulin from the second leaves of accessions ND495 (c) and TA4152-19 (d) after inoculation with *Stb16q* avirulent isolate IPO9415. The Pfafff method[6] was used to calculate relative expression values compared with time point 1 and normalization by the wheat phytochelatin gene. *Stb16q* expression and *Z. tritici* biomass were not investigated after 14 dpi in the susceptible ND495 as tissues presented necrosis. Errors bars are mean ± s.d. of the mean of three different experiments of three individual plants each. Mean Ct values were compared using the non-parametric Van der Waerden test. Group containing the same letter do not differ significantly at $P < 0.05$ according to the khi2 test. Dots represent individual data points.

a

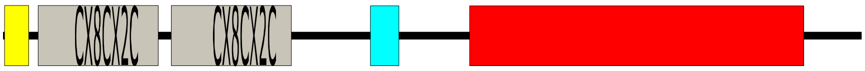

>STB16_TA4152_19
MDMVTVPLLLLLLLAPLTAADPLGQLCGNSGNYTSNSTYQANLRLLSSTLPKKAASNTNLFATATVGD
VPDMVHALALCRGDFNASVCQSCVAIAFEDAQQLCAYNKEAALYYDPCMLKFSNKNLATTDTDDKVL
LIYNTQNFTTNADITQRLLFTLINSTAQSAANSSRRFTTSRLDVGSSPTLYCLMQCTPDLTADDCMACFQ
TVLSVTLQYLGGRTGGRVLGVRCNMRYEMWPFFQGDPTLRIINLAPGVPAINNNTTPATVQPPAAAPPP
DGKATVHEQNGRSSRKRALLIIAVVAPLSSILLCFICSVVWMRRRRKGKANLNNQAATNRPEEDALVW
RLEEKSSEFTLFDFSEILHATHNFSKENLLGRGGFGPVYKGQLPDEMEIAVKRLASHSGQGFIEFKNEVE
LIAKLQHNNLVKLLGCCIQGEEKVLVYEYLPNKSLDFFIFDANRTTLVDWKKRREIIEGIAQGLLYLHKH
SRLRIIHRDLKASNILLDQDMNPKISDFGLAKIFSSNDTEGSTKRVVGTYGYMAPEYASEGIYSIKSDVFS
FGVLLLEILSGKRNSGFHQYGDFLNLLGYSWQLWEGGGWLELLEVSIVKEIHTTEARRYLNIALMCVQE
NADDRPTMSEVVAMLTSESVILPEPNHPAYSNLRVSKVHESASVVVPCSNNDVTITEEPDGR

b

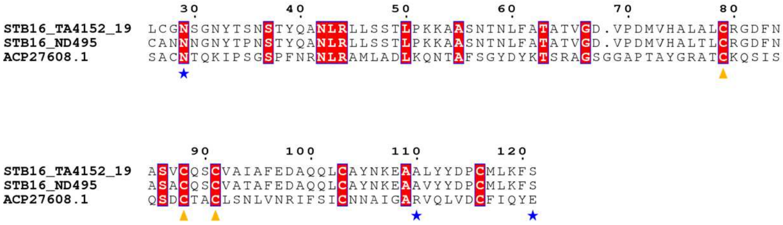

c

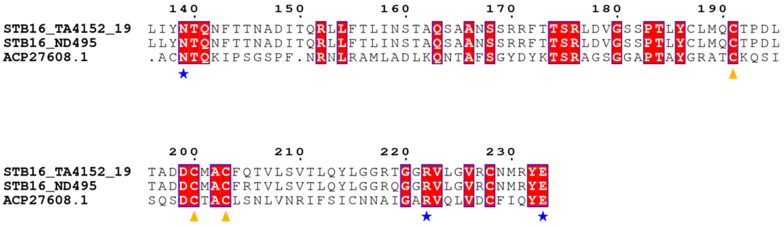

d

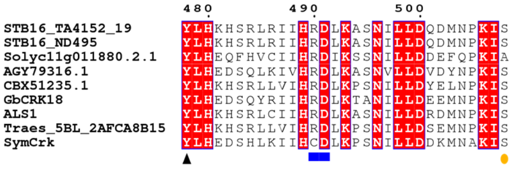

Supplementary Figure 6: STB16 amino acid sequence and domains. a/ Schematic representation and sequence of the STB16 protein. The signal peptide, DUF26 domains, the transmembrane domain and the kinase domain of STB16 are underlined in yellow, grey, blue and red, respectively. The DUF26 conserved motif CX8CX2C is depicted in both DUF26 domains. Amino acid alignment of STB16 N-terminal DUF26 (b) and STB16 C-terminal DUF26 domains (c) from resistant (TA4152-19) and susceptible (ND495) wheat accessions with the *G. biloba Gnk2* (ACP27608.1) DUF26 domain[8]. Conserved amino acids are underlined in red. Amino acids involved in *Gnk2* mannose-binding activity are marked with a blue asterisk. Cysteines of the C-X_8-C-X_2-C are indicated by orange triangles. d/Amino acid alignment of kinase activation loop of CRK which have been shown to be involved or potentially involved in interaction with microorganisms (STB16, ALS1[9], *SymCrk*[10], AGY79316.1[11], CBX51235.1[12], *GbCRK18*[13], *Solyc11g011880.2.1*[14] and *Traes_5BL_2AFCA8B15*[15])[8]. Conserved amino acids are underlined in red. The position of the phosphorylable Tyr-Val required for the immunity function of BAK1[16] is conserved in STB16 and indicated by a black triangle. The RD motif is marked by a blue bar. Amino acid change from Ser (S) to Phe (F) at position 508 and present in the EMS-derived mutant family 236 is marked by an orange circle.

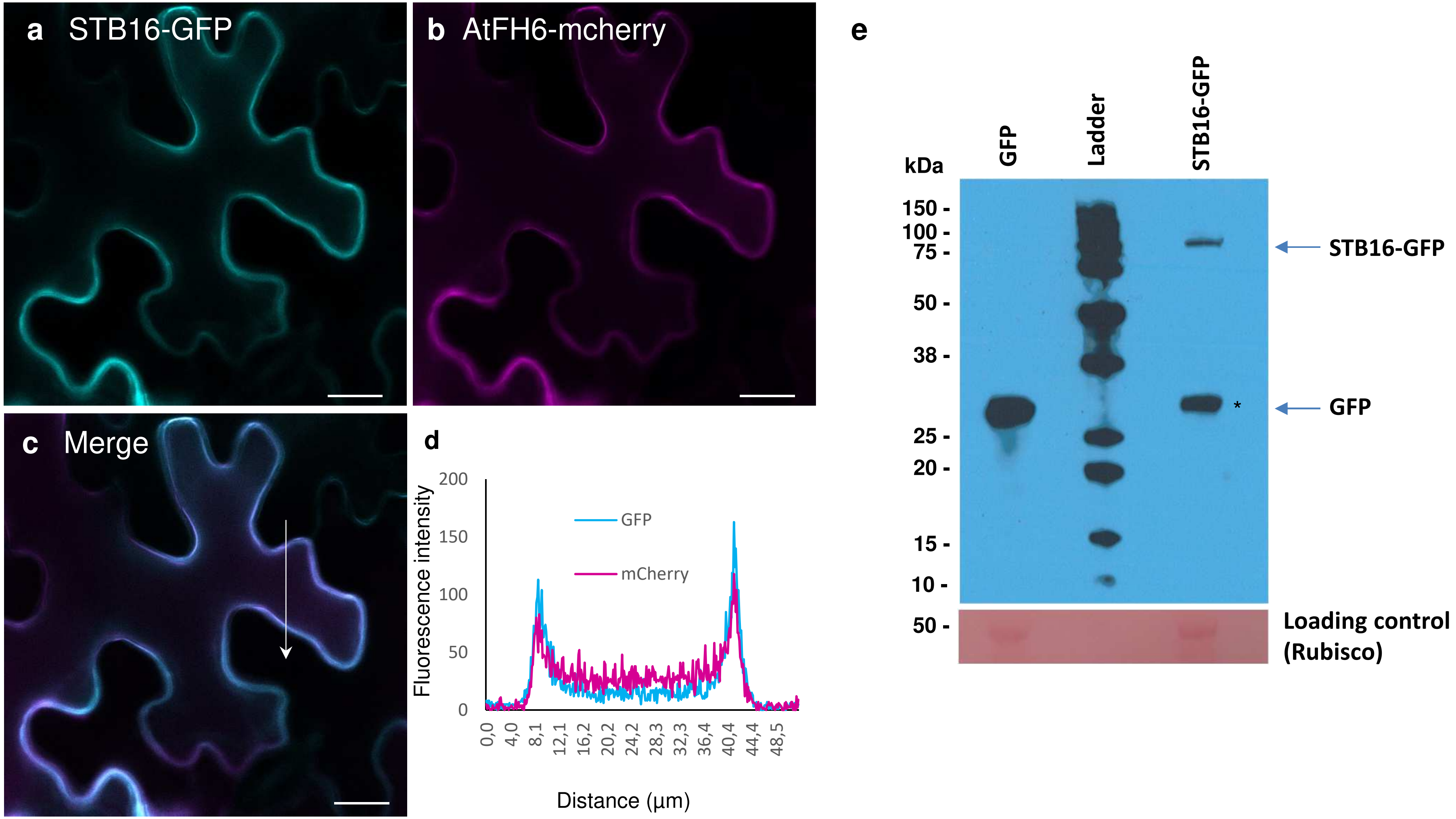

Supplementary Figure 7: Subcellular localization of STB16 in *N. benthamina* epidermal cells. a-c, Localization of the STB16-GFP (a) and the plasma membrane marker AtFH6-mCherry fusion proteins[17] (b) in *N. benthamiana* epidermal cells. **c**, merged image. Confocal images were taken 72 h post-infiltration with a LSM880 confocal microscope and processed with ZEN2.3 software. Scale bars represent 20 µm. The merged image (c) and the analysis of GFP and mCherry fluorescence intensity (arrow in c) (d) indicate that STB16 colocalize with the AtFH6 marker at the plasma membrane. e, Immunodetection of GFP and STB16-GFP proteins. Protein expression in *N. benthamiana* leaves 72 h after agro-infiltration was confirmed by immuno-blotting using an anti-GFP antibody. A cleaved GFP (*) is also observed in STB16-GFP agroinfiltrated leaves. Protein loading is indicated by Coomassie blue stain (Rubisco large subunit).

Supplementary Table 1

	IPO92006		IPO9415		IPO9455		S10		IPO2166		IPO86036		IPO90006		IPO323		IPO9699		IPO9351		LG208	
	N	P	N	P	N	P	N	P	N	P	N	P	N	P	N	P	N	P	N	P	N	P
Courtot	96	56	59	33	92	54	95	16	100	68	64	46	88	55	100	78	100	51	100	43	95	8
TA4152-19*	0	0	0	0	0	0	0	0	0	0	0	0	0	0	0	0	0	0	0	0	0	0
T2 Ct4.1.1.1*	0	0	0	0	0	0	0	0	0	0	0	0	0	0	0	0	0	0	0	0	0	0
T2 Ct4.1.1.3	98	60	60	16	84	47	85	46	100	60	50	32	83	57	100	79	100	57	70	42	94	6
T2 Ct4.2.1.3*	0	0	0	0	0	0	0	0	0	0	0	0	0	0	0	0	0	0	0	0	0	0

* lines carrying *Stb16q* (according to diagnostic marker *cfn80044*), only plants positive for the transgene were included in the analysis of T2

T2 Ct4.1.1.3 does not carry *Stb16q* and is a sister line of T2 Ct4.1.1.1

N = percentage of necrosis; P = percentage of region bearing pycnidia at 21 dpi

Supplementary Table 2

Pathogen	Race	Isolate	Resistant Plants				Susceptible Plants			
			n	Homozygous TA4152-19	Heterozygous	Homozygous CS	n	Homozygous TA4152-19	Heterozygous	Homozygous CS
Puccinia graminis	TPMK	TNMKsp1	10	2	4	4	13	2	7	4
Puccinia graminis	QFCSC	370C	5	1	3	1	21	3	9	9
Parastagonospora nodorum	NA	Sn4	13	1	10	2	2	0	2	0
Parastagonospora nodorum	NA	Sn6	17	3	10	4	0	0	0	0
Parastagonospora nodorum	NA	Sn2000	13	3	7	3	0	0	0	0
Pyrenophora tritici-repentis	1	Pti2	5	2	1	2	20	5	7	7
Pyrenophora tritici-repentis	5	DW5	10	3	5	2	15	2	8	5

Supplementary References

1. Saintenac, C., Jiang, D., Wang, S. & Akhunov, E. Sequence-Based Mapping of the Polyploid Wheat Genome. *G3: Genes|Genomes|Genetics* **3**, 1105–1114 (2013).

2. Jia, J. *et al.* Aegilops tauschii draft genome sequence reveals a gene repertoire for wheat adaptation. *Nature* **496**, 91–95 (2013).

3. Luo, M.-C. *et al.* A 4-gigabase physical map unlocks the structure and evolution of the complex genome of Aegilops tauschii, the wheat D-genome progenitor. *Proceedings of the National Academy of Sciences* **110**, 7940–7945 (2013).

4. Ramírez-González, R. H. *et al.* The transcriptional landscape of polyploid wheat. *Science* **361**, eaar6089 (2018).

5. Zadoks, J. C., Chang, T. T. & Konzak, C. F. A decimal code for the growth stages of cereals. *Weed Research* **14**, 415–421 (1974).

6. Pfaffl, M. W. A new mathematical model for relative quantification in real-time RT-PCR. *Nucleic Acids Research* **29**, 45e–445 (2001).

7. Saintenac, C. *et al.* Identification of Wheat Gene Sr35 That Confers Resistance to Ug99 Stem Rust Race Group. *Science* **341**, 783–786 (2013).

8. Robert, X. & Gouet, P. Deciphering key features in protein structures with the new ENDscript server. *Nucleic Acids Research* **42**, W320–W324 (2014).

9. Du, D. *et al.* Semi-dominant mutation in the cysteine-rich receptor-like kinase gene, *ALS 1* , conducts constitutive defence response in rice. *Plant Biology* **21**, 25–34 (2019).

10. Berrabah, F. *et al.* A nonRD receptor-like kinase prevents nodule early senescence and defense-like reactions during symbiosis. *New Phytologist* **203**, 1305–1314 (2014).

11. Yang, K. *et al.* Isolation and characterization of a novel wheat cysteine-rich receptor-like kinase gene induced by Rhizoctonia cerealis. *Scientific Reports* **3**, (2013).

12. Rayapuram, C. *et al.* Regulation of basal resistance by a powdery mildew-induced cysteine-rich receptor-like protein kinase in barley: CRK-regulated basal resistance in barley. *Molecular Plant Pathology* **13**, 135–147 (2012).

13. Li, T.-G. *et al.* Genome-Wide Identification and Functional Analyses of the CRK Gene Family in Cotton Reveals GbCRK18 Confers Verticillium Wilt Resistance in Gossypium barbadense. *Frontiers in Plant Science* **9**, (2018).

14. Yang, H. *et al.* Mapping and screening of the tomato Stemphylium lycopersici resistance gene, Sm, based on bulked segregant analysis in combination with genome resequencing. *BMC Plant Biology* **17**, (2017).

15. Ma, X., Keller, B., McDonald, B. A., Palma-Guerrero, J. & Wicker, T. Comparative Transcriptomics Reveals How Wheat Responds to Infection by *Zymoseptoria tritici*. *Molecular Plant-Microbe Interactions* **31**, 420–431 (2018).

16. Perraki, A. *et al.* Phosphocode-dependent functional dichotomy of a common co-receptor in plant signalling. *Nature* **561**, 248–252 (2018).

17. Favery, B. *et al.* Arabidopsis Formin AtFH6 Is a Plasma Membrane–Associated Protein Upregulated in Giant Cells Induced by Parasitic Nematodes. *The Plant Cell* **16**, 2529–2540 (2004).
