# Supplementary material for: A wheat cysteine-rich receptor-like kinase confers broad-spectrum resistance against Septoria tritici blotch
Source: Nat Commun. 2021 Jan 19;12:433. doi: 10.1038/s41467-020-20685-0 (PMC7815785; doi:10.1038/s41467-020-20685-0)
Supplement: Supplementary file 3 — Descriptions of Additional Supplementary Files [file 41467_2020_20685_MOESM3_ESM.pdf]

Descriptions of Additional Supplementary Files

Supplementary Data 1

Description: STB disease scores of TA4152-19. SHW accession TA4152-19 and susceptible controls (either Obelisk, Taischung or ND495) were paint-brushed inoculated with 64 different Z. tritici isolates and scored at 21 dpi.

Supplementary Data 2

Description: STB disease scores and Crk6 and Unk1 haplotypes of SHW accessions. A collection of 76 SHWs accessions and controls TA4152-19, M3 and Chinese spring were paint-brushed inoculated with five different Z. tritici isolates and scored at 21 dpi. Crk6 and Unk1 haplotypes were defined for each accession following Sanger sequencing of the first exon and the entire CDS, respectively.

Supplementary Data 3

Description: STB disease scores of M4 segregating family 236. A total of 54 M4 plants issued from a M3 plant carrying the Stb16q deleterious mutation S508F at the heterozygous state were paint-brushed inoculated with Z. tritici isolate IPO88018 and scored at 21 dpi. Genetic status of the mutation in M4 plants was determined using the KASPTM genotyping chemistry and primers cfn80052.

Supplementary Data 4

Description: Origin and Stb16q haplotypes of a hexaploid wheat collection. Haplotypes were defined following Sanger sequencing of the first Stb16q exon. Supplementary Data 5: Origin and Stb16q status of 805 wheat accessions. Presence of the Stb16q resistant allele was determined using diagnostic markers cfn80044 and cfn80045. Supplementary Data 6: Origin and Stb16q status of Aegilops tauschii accessions. Presence of the Stb16q resistant allele was determined using diagnostic markers cfn80044 and cfn80045. Supplementary Data 7: Primers used in this study.

Supplementary Data 5

Description: Origin and Stb16q status of 805 wheat accessions. Presence of the Stb16q resistant allele was determined using diagnostic markers cfn80044 and cfn80045.

Supplementary Data 6

Description: Origin and Stb16q status of Aegilops tauschii accessions. Presence of the Stb16q resistant allele was determined using diagnostic markers cfn80044 and cfn80045.

Supplementary Data 7

Description: Primers used in this study.
