# Supplementary material for: A wheat cysteine-rich receptor-like kinase confers broad-spectrum resistance against Septoria tritici blotch
Source: Nat Commun. 2021 Jan 19;12:433. doi: 10.1038/s41467-020-20685-0 (PMC7815785; doi:10.1038/s41467-020-20685-0)
Supplement: Supplementary file 11 — Reporting Summary [file 41467_2020_20685_MOESM11_ESM.pdf]

nature research

Corresponding author(s): Cyrille Saintenac

Last updated by author(s): Nov 28, 2020

Reporting Summary

Nature Research wishes to improve the reproducibility of the work that we publish. This form provides structure for consistency and transparency in reporting. For further information on Nature Research policies, see our Editorial Policies and the Editorial Policy Checklist.

Statistics

For all statistical analyses, confirm that the following items are present in the figure legend, table legend, main text, or Methods section.

n/a	Confirmed	
☐	☒	The exact sample size (*n*) for each experimental group/condition, given as a discrete number and unit of measurement
☐	☒	A statement on whether measurements were taken from distinct samples or whether the same sample was measured repeatedly
☐	☒	The statistical test(s) used AND whether they are one- or two-sided<br>*Only common tests should be described solely by name; describe more complex techniques in the Methods section.*
☒	☐	A description of all covariates tested
☒	☐	A description of any assumptions or corrections, such as tests of normality and adjustment for multiple comparisons
☐	☒	A full description of the statistical parameters including central tendency (e.g. means) or other basic estimates (e.g. regression coefficient) AND variation (e.g. standard deviation) or associated estimates of uncertainty (e.g. confidence intervals)
☐	☒	For null hypothesis testing, the test statistic (e.g. *F*, *t*, *r*) with confidence intervals, effect sizes, degrees of freedom and *P* value noted<br>*Give P values as exact values whenever suitable.*
☒	☐	For Bayesian analysis, information on the choice of priors and Markov chain Monte Carlo settings
☒	☐	For hierarchical and complex designs, identification of the appropriate level for tests and full reporting of outcomes
☒	☐	Estimates of effect sizes (e.g. Cohen's *d*, Pearson's *r*), indicating how they were calculated

Our web collection on statistics for biologists contains articles on many of the points above.

Software and code

Policy information about availability of computer code

Data collection	Confocal images used to visualize STB16 subcellular localization and pathogen infection were acquired using the Zeiss ZEN2.3 SP1 (black edition) version 14.0.7.201 and the Zeiss Zen Black 2012 softwares, respectively.
Data analysis	Primers were designed using the Primer3Plus software and microsatellite identified using SSRLocator v1.0.<br>SSR Genotyping data were anaylsed using the GenMapper software version 5.0.<br>All CEL files from the GeneTitan were analysed with the APT suite (Analysis Power Tools, version 1.20.0)<br>Genetic maps were derived from RQLpackage version 1.29 and Carthagene-1.3.beta.<br>The Basic Local Alignment Search Tool (BLAST) version 2.2.26 was used to compare sequences.<br>Assembly of PacBio reads was performed using the SMRT® Analysis (v2.2.0) software.<br>BAC sequence annotation was performed using the TriAnnot software pipeline.<br>PROVEAN version 1.1.5 was used to analyze the impact of CRK6 mutation.<br>KASP markers and real-time expression data were analyzed using the LightCycler 480 version 1.5.1 Roche Life Science.<br>Confocal images for STB16 subcellular localization and pathogen infection were analyzed using the ZEN 2.3 (blue edition) version 2.2.69.1000 and Fiji package of ImageJ version 2.0.0-rc-69/1.52p, respectively.<br>Stb16q sequences were analysed using the MEGA sotware version 5.1.<br>RStudio version 1.3.1093 (R version 3.4.4) was used to perform statistical analysis.

For manuscripts utilizing custom algorithms or software that are central to the research but not yet described in published literature, software must be made available to editors and reviewers. We strongly encourage code deposition in a community repository (e.g. GitHub). See the Nature Research guidelines for submitting code & software for further information.

Data

Policy information about availability of data

All manuscripts must include a data availability statement. This statement should provide the following information, where applicable:

- Accession codes, unique identifiers, or web links for publicly available datasets
- A list of figures that have associated raw data
- A description of any restrictions on data availability

The source data for Supplementary Figures 5 and 7, and Supplementary Tables Data 1, 2 and Supplementary Tables, 1 and 24 and 8 are provided as a source data file. Stb16q haplotypes and BAC clones sequences are available as GenBank accession numbers MT231554-231565 [https://www.ncbi.nlm.nih.gov/nuccore/MT231554-231565], MT932490 [https://www.ncbi.nlm.nih.gov/nuccore/MT932490], MT942596 [https://www.ncbi.nlm.nih.gov/nuccore/MT942596.1/] and MT942597 [https://www.ncbi.nlm.nih.gov/nuccore/MT942597]. BAC clones raw data are available as SRA number PRJNA680685 [https://www.ncbi.nlm.nih.gov/sra/PRJNA680685].

Field-specific reporting

Please select the one below that is the best fit for your research. If you are not sure, read the appropriate sections before making your selection.

☒ Life sciences ☐ Behavioural & social sciences ☐ Ecological, evolutionary & environmental sciences

For a reference copy of the document with all sections, see nature.com/documents/nr-reporting-summary-flat.pdf

Life sciences study design

All studies must disclose on these points even when the disclosure is negative.

Sample size	At least 3 but more generally 6/10 biological replicates were performed. No sample-size calculation was performed. Sample sizes were selected according to previous experiments (Saintenac et al., 2018, Nature Genetics) and according to our knowledge of phenotypic variability for each experiment. Unlike quantitative resistance, Stb16q is considered as a major resistance gene meaning that it has a major effect on the phenotype. We considered that sample sizes used were enough to conclude on the presence or not of the gene.
Data exclusions	No data were excluded from the analysis.
Replication	All experiments were replicated at least three times and all replicates were successful.
Randomization	Individual plants were randomly distributed in growth chamber for each phenotyping and expression experiment.
Blinding	Investigators were not blinded to group allocation for phenotyping transgenic individuals carrying or not the Stb16q gene. The phenotypic difference between both groups (carrying or not the gene) was very important which did not require blinding.

Reporting for specific materials, systems and methods

We require information from authors about some types of materials, experimental systems and methods used in many studies. Here, indicate whether each material, system or method listed is relevant to your study. If you are not sure if a list item applies to your research, read the appropriate section before selecting a response.

Materials & experimental systems

n/a	Involved in the study	
☐	☒	Antibodies
☒	☐	Eukaryotic cell lines
☒	☐	Palaeontology and archaeology
☒	☐	Animals and other organisms
☒	☐	Human research participants
☒	☐	Clinical data
☒	☐	Dual use research of concern

Methods

n/a	Involved in the study	
☒	☐	ChIP-seq
☒	☐	Flow cytometry
☒	☐	MRI-based neuroimaging

Antibodies

Antibodies used	Primary rat monoclonal GFP antibodies at 1:5,000 (#3H09, lot no. 60706001AB, isotype IgG2a, ChromoTek, Martinsried, Germany)<br>Secondary antibodies goat anti-rat IgG-HRP at 1:10,000 (AS10 1187, lot no.1710, Agrisera Sweeden)
Validation	As presented in the manufacter website, the GFP-Antibody 3H9 was independently validated for Western Blotting by Moores Cancer Center, UC San Diego (No. #029762; Date 07/07/2014; https://www.chromotek.com/fileadmin/content/Images/Antibodies__IgGs/GFP_3H9/029762_validation_report_Chromotek.pdf).

In addition, validations in transient expression in Nicotiana benthamiana leaves were previously published (e.g. Mair et al. eLife 2019;8:e47864; see Figure 1; https://elifesciences.org/articles/47864#s2). No major bands were observed in the negative control (untransformed N. benthamiana cells).
